# The Effect of Surfactant Content over Cu-Ni Coatings Electroplated by the sc-CO_2_ Technique

**DOI:** 10.3390/ma10040428

**Published:** 2017-04-19

**Authors:** Ho-Chiao Chuang, Jorge Sánchez, Hsiang-Yun Cheng

**Affiliations:** Department of Mechanical Engineering, National Taipei University of Technology, Taipei 10608, Taiwan; t103569010@gmail.com (J.S.); mark155214155214@yahoo.com.tw (H.-Y.C.)

**Keywords:** supercritical CO_2_, electroplating, 1,4-butynediol, Cu-Ni coating, internal stress, electrochemical resistance

## Abstract

Co-plating of Cu-Ni coatings by supercritical CO_2_ (sc-CO_2_) and conventional electroplating processes was studied in this work. 1,4-butynediol was chosen as the surfactant and the effects of adjusting the surfactant content were described. Although the sc-CO_2_ process displayed lower current efficiency, it effectively removed excess hydrogen that causes defects on the coating surface, refined grain size, reduced surface roughness, and increased electrochemical resistance. Surface roughness of coatings fabricated by the sc-CO_2_ process was reduced by an average of 10%, and a maximum of 55%, compared to conventional process at different fabrication parameters. Cu-Ni coatings produced by the sc-CO_2_ process displayed increased corrosion potential of ~0.05 V over Cu-Ni coatings produced by the conventional process, and 0.175 V over pure Cu coatings produced by the conventional process. For coatings ~10 µm thick, internal stress developed from the sc-CO_2_ process were ~20 MPa lower than conventional process. Finally, the preferred crystal orientation of the fabricated coatings remained in the (111) direction regardless of the process used or surfactant content.

## 1. Introduction

Cu and Cu-rich alloys are popular engineering materials due to their many technological and metallurgical uses. However, Cu is a relatively soft metal with poor chemical properties for some applications, so efforts are being made to solve these issues. One such solution is alloying Cu with Ni; the addition of even small quantities of Ni effectively enhances the mechanical and chemical properties of pure Cu. The earliest research reported for this topic was on Monel alloys [1], which present good ductility and thermal conductivity. Cu-Ni alloys are important engineering materials due to their characteristic mechanical, magnetic, electrochemical, and electrocatalytic properties [2]. Nowadays, many industrial applications rely on Cu-Ni alloys; they are desirable for highly-corrosive environments, such as marine and microbiological applications. Moreover, resistance to pitting corrosion, crevice corrosion, and stress corrosion is desired for high-temperature applications [3]. Cu-Ni alloy coatings with various compositions are used in the microelectronics industry and in portable electronics such as integrated circuits, personal computers, and cellphones. However, an issue with Cu-Ni co-plating is the relatively large difference in electric potential for chemical reduction, ranging from +0.35 vs. a standard hydrogen electrode (SHE) to −0.25 vs. SHE. To perform direct current (DC) electroplating with no surfactants, a relatively higher current density is required. Consequently, increasing current density results in surface defects, causing different issues. Thus, efforts should be focused on the improvement of the electroplating process at low DC settings.

Baskaran et al. [4] performed experiments using pulse electroplating, adjusting the current density, and studied the effects of heat treatment over their samples; they found that the composition of Cu-Ni alloy could be affected by changes in current density applied during electroplating. Alper et al. [5] reported electroplating using a three-electrode setup supplying negative potential and adjusting pH values, which affected the surface morphology and magnetic properties of the samples. Desislava et al. [6] studied the Hull cell electroplating method, discussing the differences in surface morphology and the degree of influence over the grain size by adjusting Cu and Ni ion concentrations in solution and the current density. Moreover, it has been shown that additives can change the properties of coatings and some even reduce the internal stress in thin films [7].

1,4-butynediol is an additive frequently used to obtain metal deposits with fine grains, bright surfaces, and it provides a levelling effect under the Watt’s bath electrolyte process [8]. Atanassov et al. [9] studied the effect of butynediol over Ni coatings. 1,4-butynediol contain hydroxyl groups, OH, which can act as a base towards H^+^ ions, reacting with them in solution, allowing the cathode to produce a negative shift in potential. Consequently, Ni was co-plated with Cu. Additionally, as a type of gemini surfactant, 1,4-butynediol, with an ideal solution interface adsorption capacity, can depress rapid accumulation of H^+^ and simultaneously promote the migration rate of H^+^ from the cathode to the solution interface [10]. 

Even though adding 1,4-butynediol makes the plated surfaces smoother, the conventional process showed a disadvantage when the sample surface has a complex morphology. This reduces the ability of the electrolyte to flow smoothly, increases viscosity, and creates residual H^+^ in the coating during electroplating. Residual H^+^ can create defects, such as pinholes [11], which increase surface roughness, internal stresses, and reduces corrosion resistance. Some studies have reported sc-CO_2_ mixed with common electroplating solution, resulting in coatings with the following characteristics: (1) finer grains; (2) smoother surfaces; (3) increased wear resistance; and (4) increased corrosion resistance [12,13,14,15,16]. Sc-CO_2_ electroplating for both Cu and Ni have become increasingly popular in the past decades for fabrication of coatings and structures because of superior coverage capabilities and enhanced mechanical properties provided to the electroplated metal [12,17]. The introduction of additives, such as 1,4-butynediol, to the electrolyte and the sc-CO_2_ electroplating process both present advantages over the conventional electroplating process for the deposition of Cu-Ni coatings, however, there is no reports in the literature discussing co-plating of Cu-Ni alloys under sc-CO_2_ electroplating with the addition of 1,4-butynediol. 1,4-butynediol can be considered an effective surfactant in this context, because by reducing localized concentrations of H^+^ in solution it promotes emulsification between the sc-CO_2_ and electrolyte.

In this work, 1,4-butynediol was introduced to the sc-CO_2_ electroplating process to reduce both internal stresses and surface roughness, and to study the co-plating of Cu-Ni coatings under a pressure of 15 MPa and temperature of 50 °C. Various analyses of mechanical and chemical properties were performed, which will be described in more detail in the sections below.

## 2. Experimental

### 2.1. Materials and System Configuration

The anode used in this study was a piece of Pt with dimensions 20 mm × 20 mm × 0.2 mm. The cathode used in this study was a circular slab of brass with a diameter of 2.45 cm and thickness of 0.5 mm. The electrolyte was produced in the laboratory and composed of 0.8 M nickel (II) sulfate hexahydrate (NiSO_4_·6H_2_O), 0.2 M copper (II) sulfate pentahydrate (CuSO_4_·5H_2_O), and 0.28 M citric acid (C_6_H_10_O_8_). 1,4-butynediol (C_4_H_6_O_2_) content was adjusted to 2.0 g/L, 2.5 g/L, 3.0 g/L, 3.5 g/L, and 4.0 g/L. 

**Figure 1:**
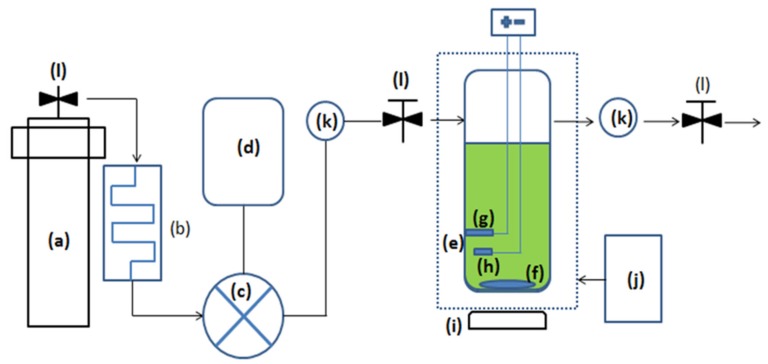
Schematics of supercritical electroplating equipment: (**a**) CO_2_ tank; (**b**) heat exchange device; (**c**) high pressure pump; (**d**) compressor; (**e**) hot water lining; (**f**) magnetic stir bar; (**g**) anode; (**h**) cathode; (**i**) magnetic stirrer machine; (**j**) thermostatic water tank; (**k**) pressure gages; and (**l**) pressure valves.

The electroplating methods depicted in this work were performed in a high-pressure electroplating chamber, purchased from Tech Lead Enterprise Co. Ltd. (New Taipei City, Taiwan); schematics are shown in Figure 1. The chamber is made of SAE 316 grade stainless steel; the inner wall of the chamber is covered by a Teflon liner to protect against corrosion from the electrolyte; a stir bar covered in Teflon is placed inside the chamber, and the magnetic stirrer machine is on the outside of the chamber to mix the sc-CO_2_ with the electrolyte; after closing the chamber, the internal capacity was 180 mL. The supercritical fluid used in this study was CO_2_ gas with 99.9% purity, purchased from C.C. Gaseous Co. (New Taipei City, Taiwan). The CO_2_ tank is connected to a heat exchange device that cools the gas to liquefied form. The volumetric CO_2_ concentration was around 30%. A DC power supply, model GDP-3303S, purchased from GW Instek (New Taipei City, Taiwan), was used to perform the electroplating. 

### 2.2. Sample Preparation and Experimental Method

Brass samples were ground with sandpaper grit P800, P1500, and P2000 and sequentially polished to a mirror finish with alumina powder with particulate sizes of 1 µm, 0.3 µm, and 0.05 µm to remove surface oxide from the surface. Samples were then placed inside a glass beaker where isopropyl alcohol (IPA) was poured. The glass beaker was then placed inside an ultrasonic cleaner for five minutes. Next, IPA was poured out and acetone was poured into the glass beaker. Then it was placed again in the ultrasonic cleaner for five minutes. Finally the sample was rinsed with de-ionized (DI) water, blown dry with nitrogen gas and placed in a dry cabinet to avoid contamination. Before electroplating, one side of the sample was covered with PVC anti-plating tape. Then samples underwent pre-treatment: they were immersed in 2% NaOH solution to remove organic contaminants from the surface, and then in 35% HCl solution to activate the surface (15 s each); the sample was then rinsed in DI water, blown dry with nitrogen gas and, finally, the sample was ready for electroplating. 

The sample was placed inside the high-pressure electroplating chamber, where liquefied CO_2_ was pumped in; temperature and pressure were adjusted to achieve optimal sc-CO_2_ electroplating parameters (pressure: 15 MPa, temperature: 50 °C) [18,19]. Magnetic agitation was started at 500 rpm for 30 minutes to allow homogenous mixing between the sc-CO_2_ and electrolyte, then kept constant throughout the remainder of the experiment. The power supply was then connected, the desired DC values adjusted and, finally, electroplating was performed. The desired coating thickness was 50 µm. After electroplating was finished, the pressure was released to open the chamber, the sample retrieved and soaked in DI water to rinse off any remnants of electrolyte from the surface. The sample was then blown dry with nitrogen gas and stored for future analysis. 

For conventional electroplating, samples underwent similar pre-treatment as described above. However there were no pressure pumping or mixing steps after the samples were placed inside the chamber. Temperature and agitation were adjusted to 50 °C and 500 rpm, respectively, the power supply connected with the desired DC values adjusted, and electroplating was performed. The electroplating parameters are listed in Table 1.

### 2.3. Current Efficiency Analyses

When electrical current passes through the electrolyte, the anode and cathode, respectively, undergo oxidation and reduction simultaneously, converting electrical energy into chemical energy. Cathode reactions are accompanied by hydrogen evolution, meaning current was not completely used for reduction. The current efficiency of the Cu-Ni electroplating is calculated by Equation (1) shown below:

(1) $$\Psi =\left(\frac{2\cdot F}{I\cdot t}\times 100\right)\cdot \left(\frac{W_{Ni}}{58.69}+\frac{W_{Cu}}{63.54}\right),$$

where ψ represents current efficiency (%), *F* represents the Faraday constant (96,500 C/mol), *I* is the electrical current (A), and *t* is the electroplating time (s); *W_Ni_* represents the weight of the Ni deposited at the cathode, *W_Cu_* represents the weight of the Cu deposited at the cathode (both calculated by ion concentration in solution). The number of electrons needed for Cu-Ni ion reduction is 2.

### 2.4. Microstructural Analyses

The composition analysis of the coatings in this study was performed with a high-resolution SuperProbe Electron Probe Micro Analyzer (EPMA) model JXA-8200 by JEOL, Ltd. (Tokyo, Japan). The crystalline structure was measured by X-ray diffractometer model M03XHF by MAC Science (Yokohama, Japan), under a scanning speed of 2 deg/min, and the grain size was calculated from the main diffraction peaks by the Scherrer equation [20]. For grain size calculations, additional factors for line broadening were not considered and the results achieved are used as qualitative descriptions of the trends rather than a precise quantitative tool. Precise measurements should consider the effects of micro-twins, stacking faults, uniform stresses, etc. Microstructural surface morphology was observed by field emission scanning electron microscope (FESEM), model Σigma Essential by Zeiss Microscopy GmbH (Jena, Germany). Surface roughness (R_a_) was measured using α-step profilometer model Surfcoder SEF3500 by Kosaka Laboratory Ltd. (Tokyo, Japan) and interpreted by the accompanying software.

### 2.5. Internal Stress Analyses

Internal stresses can seriously affect the quality of coatings. Internal stresses in the coatings form due to unbalanced crystallization during the plating process. There are five main factors in the creation of internal stresses: (1) crystalline joining; (2) hydrogen penetration; (3) co-plating of foreign substances; (4) excess energy; and (5) lattice defects [21].

For this work, internal stresses were calculated using a laser displacement setup to measure the bending of substrates after electroplating. The substrate used for this analysis was a rectangular slab of Cu with dimensions 30 mm × 7 mm × 0.1 mm. Samples were placed on top of an XY moving table, where a single point was measured at a time. The laser sensor shoots a beam at the surface to determine differences in height relative to a reset point. The data were then introduced into SigmaPlot^®^ software (Version 11.0, Systat Software Inc., San Jose, CA, USA) to plot a graph, which was curve-fitted by linear regression to calculate the radius of curvature (R); R was then inserted into the Stoney equation to calculate internal stresses, as shown in Equation (2) below:

(2) $$\sigma_{f}=\frac{E_{s}\cdot t_{s}^{2}}{6(1-\upsilon_{s})Rt_{f}},$$

where *σ_f_* is the calculated internal stress in the coating; *E*_s_ and *υ*_s_ are the material’s Young’s modulus and Poisson ratio, respectively; *R* is the calculated radius of curvature; *t_s_* and *t_f_* are the thicknesses of the substrate and coating, respectively. Quantification of internal stresses by this method has been previously reported [22].

### 2.6. Electrochemical Analyses

In this work electrochemical analysis was performed via potentiodynamic polarization scanning (PPS) to obtain anodic and cathodic polarization curves for each parameter. Electrochemical experiments were performed using a potentiostat (model ECW-5000, Jiehan Technology Corp., Taichung, Taiwan) with the produced coating serving as working electrode (WE), a piece of graphite as counter electrode, and a saturated calomel electrode (SCE) as reference. Experimental parameters are shown in Table 2.

The WE was submerged into a corrosive solution and allowed to stabilize for 30 min to obtain the open circuit potential (OCP). OCP was assumed to be the corrosion potential (E_corr_) and a suitable range for PPS is set from E_corr_. High E_corr_ represents low chemical reactivity, and vice versa. Through PPS, a polarization curve containing both cathodic and anodic polarization curves was obtained. From Tafel extrapolation by Cview software (Version 2.6b, Scribner Associates Inc., Southern Pines, NC, USA), the corrosion current density (I_corr_) can be calculated. Finally, from the I_corr_ we can estimate the material corrosion rate. Measurement of the electrochemical properties by this method was reported in a previous study [22]; theoretical concepts of Tafel analysis and examples of typical polarization curves can be found in the literature [23].

## 3. Results and Discussion

### 3.1. Current Efficiency (CE) Analyses

**Figure 2:**
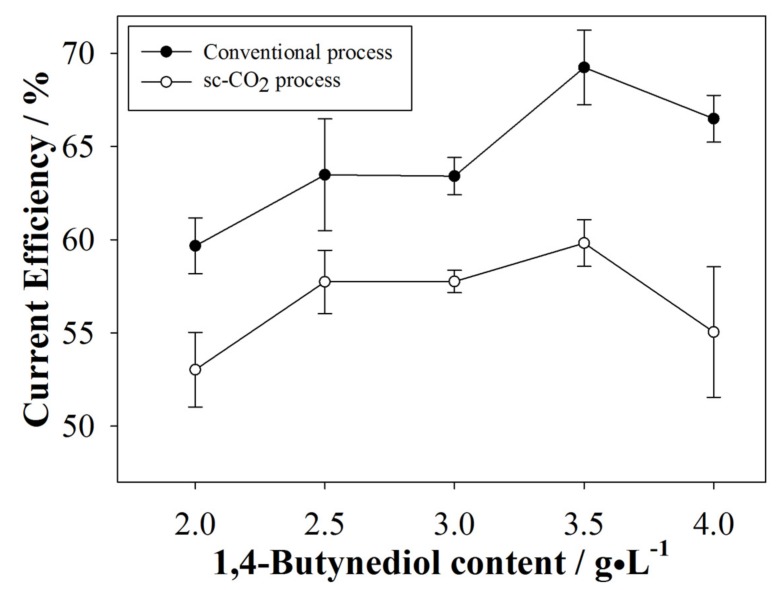
Comparison of the CE between the conventional and sc-CO_2_ electroplating.

The CE was calculated by measuring the sample’s weight before and after electroplating. Results are shown in Figure 2. The CE of the conventional process was ~65%, and the CE of the sc-CO_2_ process was approximately 56%. Secondary reactions waste electrical charge, reducing the entire process efficiency [24], however, the CE of the conventional process was ~10% higher than the sc-CO_2_ process due to dissolved CO_2_ in solution, which lowers the CE even further. However, CE was evidently improved by the addition of even a small quantity of 1,4-butynediol for both processes. 1,4-butynediol is known to undergo hydrogenation under a sc-CO_2_ environment [25], so it is possible that the surfactant could bind with extra H^+^ that initially reduced the current efficiency of the plating processes. Furthermore, a steady flat line was achieved when increasing the surfactant content to 3.0 g/L; we believe this is a sign of quasi-saturation of the surfactant in solution. Further increasing the surfactant content might overcome the saturation condition and help to absorb H^+^ again. However, after increasing surfactant content to 4.0 g/L and over, steady drop of current efficiency was observed; further increases of surfactant content did not significantly affect the current efficiency, which could be attributed to the inability of the electrolyte to dissolve more surfactant after achieving complete saturation.

### 3.2. Microstructural Analyses

#### 3.2.1. Nickel Content of the Plated Coatings

**Figure 3:**
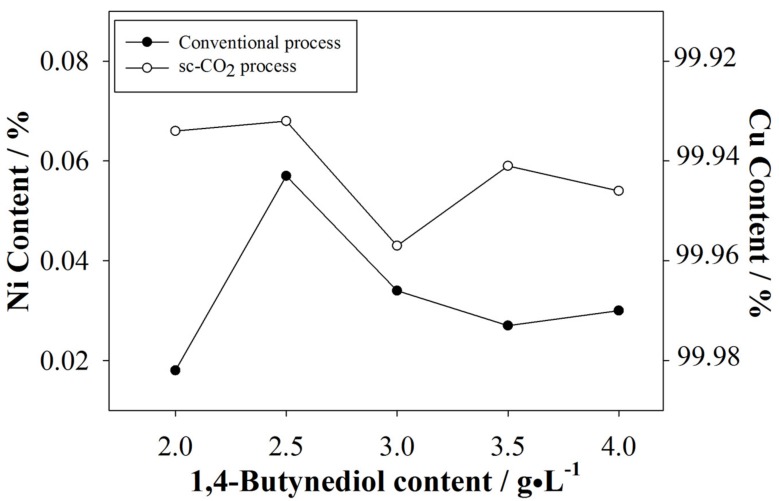
Comparison of Ni content between the conventional and sc-CO_2_ electroplating.

The elemental contents and composition of the plated coatings as measured by EPMA are shown in Figure 3. Since it is a costly and time-consuming analysis, EPMA measurement was performed only once per sample. As stated above, the addition of 1,4-butynediol to the emulsified electrolyte reduced excess H^+^ in solution, disabling their ability to remain at the cathode surface; therefore, the produced coatings were relatively more densely packed. This becomes evident from SEM observations which will be discussed later (see details in Section 3.2.3). From Figure 3 we also infer real surfactant saturation was achieved at 2.5 g/L, because further increasing the surfactant content no longer significantly affected the Ni content on the coatings.

#### 3.2.2. Average Grain Size of the Plated Coatings

**Figure 4:**
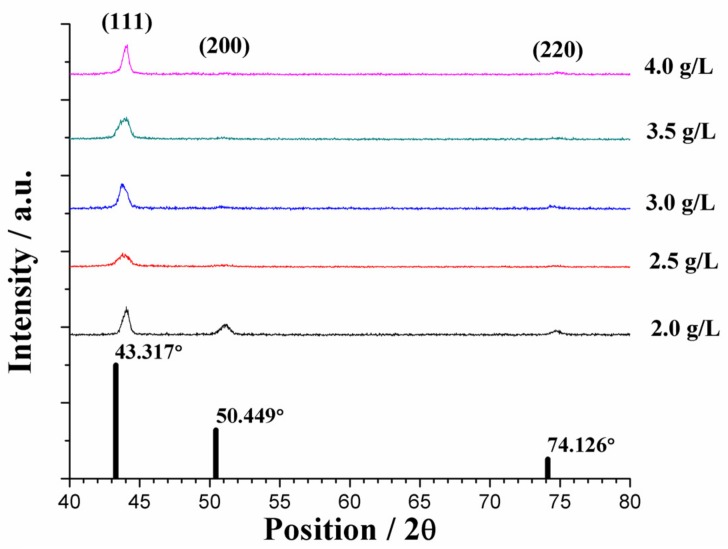
XRD peaks of the conventional process under various surfactant content levels. The reference patterns of elemental Cu (ICDD# 01-085-1326) are shown as black bars.

**Figure 5:**
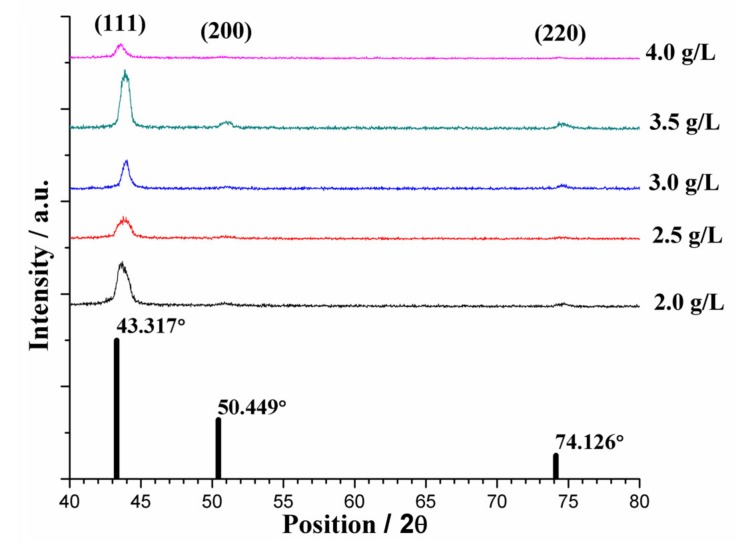
XRD peaks of the sc-CO_2_ process under various surfactant content levels. The reference patterns of elemental Cu (ICDD# 01-085-1326) are shown as black bars.

The usual factors that affect the deposition mechanism include applied current density, Ni ion concentration, and surfactant content in the electrolyte. Increases in current density are known to effectively decrease crystallite size, and vice versa. Ni ion concentration is known to affect polarization because it provides enhanced mass transfer [6]. The sc-CO_2_ environment is also known to affect the deposition mechanism due to the effect similar to pulse plating [14]. However, for this specific study, current density and Ni ion concentration remained fixed throughout the experiment, reducing the number of affecting factors. The sc-CO_2_ parameters chosen were the optimal parameters found from previous studies [17,18,19,22], thus, their deposition mechanism was understood. Differences in surfactant content are seen to affect the crystalline structure of coatings, shifting the intensity of the measured peaks. Crystallographic orientations were measured by X-ray diffraction (XRD) for sc-CO_2_ and conventional processes and are shown, respectively, in Figure 4 and Figure 5. For both cases the strongest peak was in the (111) orientation located at ~43°, with slight signals in the (200) and (220) directions, evidence of face-centered cubic (fcc) structures. Since the produced coatings had an overall higher Cu content compared to Ni, they were compared to an elemental Cu XRD reference pattern. Regardless of the electroplating process or surfactant content, the preferred orientation remained unchanged. No detectable peaks of impurities were found. However, the shifts observed from the elemental Cu pattern to the measured XRD patterns were most likely due to co-plating with Ni.

**Figure 6:**
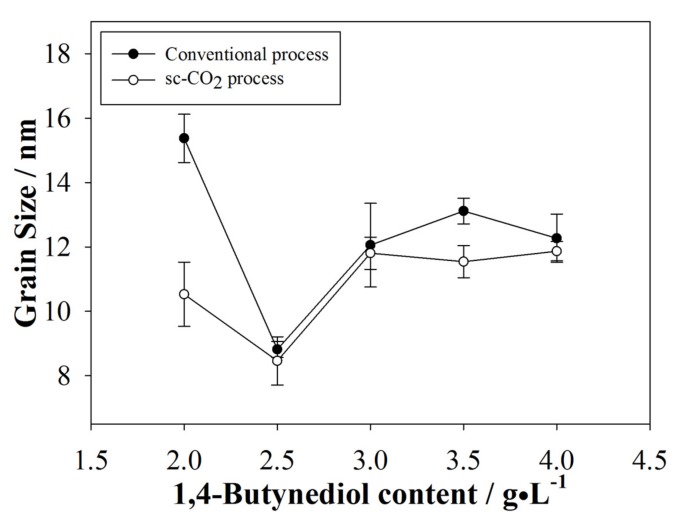
A comparison of the grain size between the conventional and sc-CO_2_ processes.

Coatings produced by the sc-CO_2_ process were expected to have smaller grain sizes and, indeed, peak broadening was observed from coatings produced by the sc-CO_2_ process, hinting to the relatively smaller grain sizes. The calculated results are shown in Figure 6. The sc-CO_2_ process under optimal parameters should always result in coatings with smaller grain sizes compared to the conventional process. Extra H^+^ in the solution is attracted to the substrate surface carrying the negative potential, which causes momentary passivation of the substrate’s surface; thus, no electroplating occurs. Metal ions attach to the substrate surface and commence nucleation. The sc-CO_2_ mixed with the electrolyte reacts with the extra H^+^ forming carbonic acid, and effectively removes H^+^ from the substrate surface. New metal ions can attach to the substrate’s surface. These processes occur continuously, resulting in alternating on-off periods. At the chosen pressure, plating on time is short and leads to higher surface concentration of the metal ions, restricting grain growth. 1,4-butynediol is also known to react with hydrogen at high pressures [26]; thus, more H^+^ could be removed from the substrate surface. Increasing surfactant content affects the on-off periods, restricting grain size even further. However, on-off periods were disturbed over the threshold of 2.5 g/L and, thus, we see from Figure 6 that the grain size increased once again.

#### 3.2.3. Surface Morphology of the Plated Coatings

**Figure 7:**
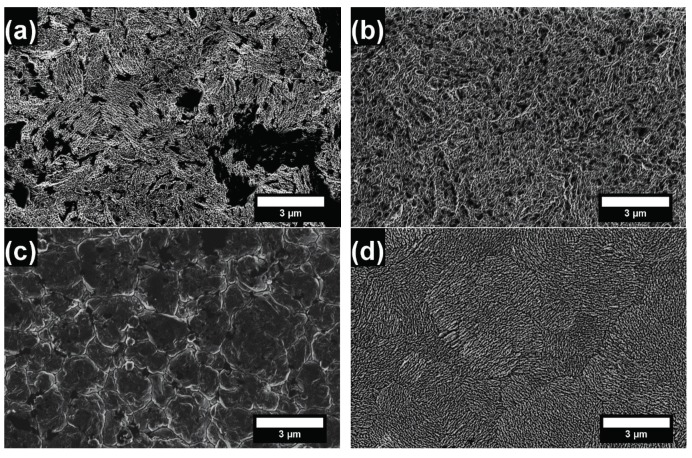
Representative SEM images of coating surface morphologies fabricated by the conventional and sc-CO_2_ processes at different surfactant content levels: (**a**) conventional and (**b**) sc-CO_2_ processes with 2.0 g/L 1,4-butynediol; and (**c**) the conventional and (**d**) sc-CO_2_ process with 2.5 g/L 1,4-butynediol.

Coating surface morphologies plated by conventional and sc-CO_2_ processes were observed by FESEM. Representative figures that show the effects of the process and surfactant concentration were selected and are shown in Figure 7. Coatings produced by the sc-CO_2_ process were smoother than those produced by the conventional process and presented fewer defects. The addition of the surfactant is able to reduce excess H^+^ that would otherwise attach to the substrate’s surface. Sc-CO_2_ is known to possess high solubility with hydrogen, so it was expected that surface morphology in the sc-CO_2_ process becomes smoother for near-saturation concentrations, and vice versa. Figure 7a shows the film produced by the conventional method with 2.0 g/L of surfactant. The morphology is random and uneven with visible dark patches. However, this is not as evident in Figure 7b, which shows the film produced by the sc-CO_2_ method with 2.0 g/L of surfactant. There were no large, dark patches, suggesting enhanced coverage of the surface. Dark patches in SEM images represent areas with poor adhesion where the coating has peeled off (thought to be the case in Figure 7a) or areas of low conductivity (such as oxides) which do not allow electrons to bounce back to the SEM detector properly, similar to the case of Figure 7b.

Figure 7c shows the film produced by the conventional method with 2.5 g/L of surfactant, which was the optimal concentration for many of the characteristics described previously. It is clearly observed that the crystal growth was more ordered and structured. This could be attributed to the reduction of excess H^+^ in the electrolyte. Moreover, Figure 7d shows the film produced by the sc-CO_2_ process with 2.5 g/L of surfactant, which displayed the smallest grains observed in this study. At similar scales, the size of grains is clearly smaller in the coating produced by the conventional process or in those produced by the sc-CO_2_ process with various surfactant contents, which supports the results observed from Figure 6. In contrast, excess H^+^ was absorbed relatively easily by the substrate surface in conventional plating, creating more pinholes and defects.

#### 3.2.4. Surface Roughness of the Plated Coatings

**Figure 8:**
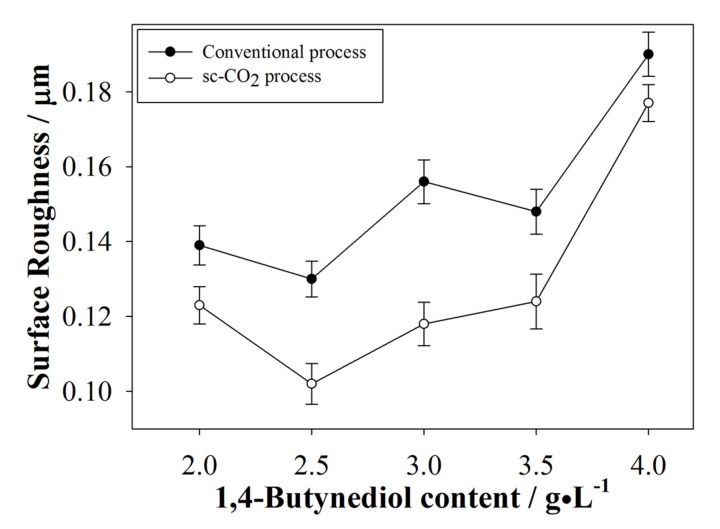
Comparison of surface roughness between the conventional and sc-CO_2_ processes.

Average Ra was measured by an α-step profilometer and the results are shown in Figure 8. From Figure 7 it was seen that morphology of coatings produced by the conventional process contained relatively more defects, thus, the average Ra is expected to be higher than for coatings produced by the sc-CO_2_ process. Roughness of coatings with near saturation surfactant content are expected to be lower and, indeed, a trend similar with the results described above was observed.

### 3.3. Internal Stress of the Plated Coatings

**Figure 9:**
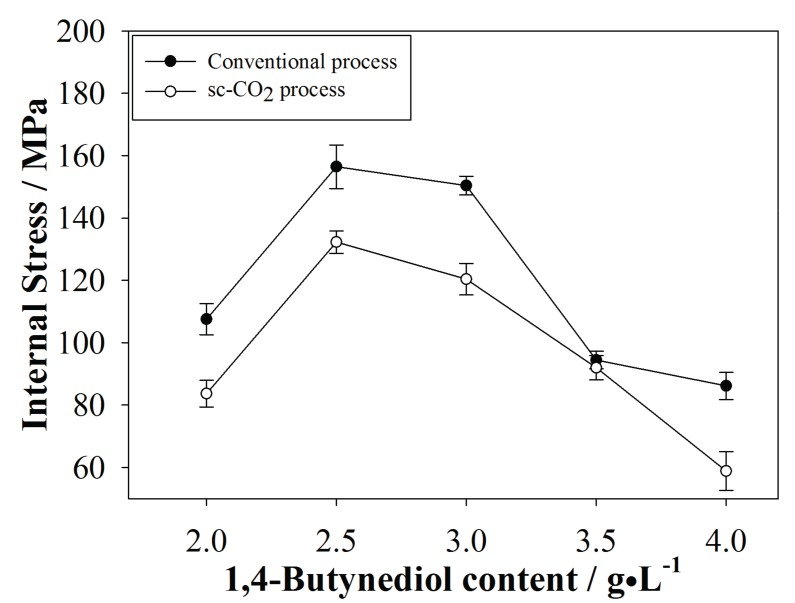
Internal stresses calculated from coatings fabricated by the conventional and sc-CO_2_ processes.

The calculated internal stresses of coatings produced in this study are shown in Figure 9. The internal stresses found in coatings produced by the sc-CO_2_ process were lower than those found in the conventional process. During the conventional process, excess H^+^ in solution attached to the substrate surface, leaving behind voids and compressive stresses after electroplating was completed. In the sc-CO_2_ process, CO_2_ in the solution removed excess H^+^ from the surface, effectively reducing voids and internal stresses. Additionally, we observe a similar, but inverse, relationship between surfactant content with internal stress relative to that with grain size, which is expected. Reduced grain size should, indeed, present increased internal stresses; however, addition of surfactant with the sc-CO_2_ process generally reduced internal stress compared to the conventional process. Moreover, we infer that the compositions, crystal structures, and local orientations were inconsistent when co-plating different metals, changing the volume of the coating, and exerting influence over the degree of internal stresses.

### 3.4. Electrochemical Properties of the Plated Coatings

**Figure 10:**
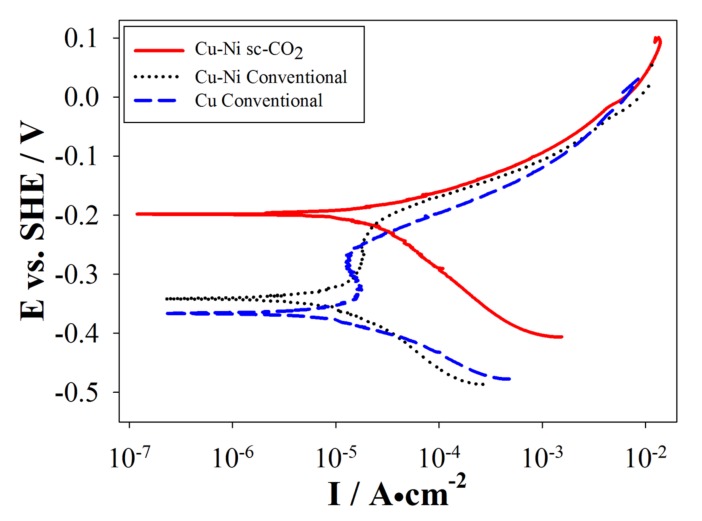
Potentiodynamic polarization curve of a sample fabricated with 2.5 g/L surfactant content.

**Figure 11:**
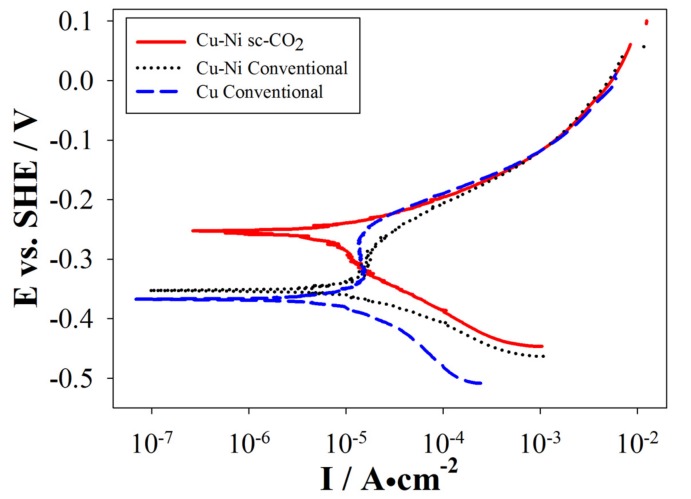
Potentiodynamic polarization curve of a sample fabricated with 3.0 g/L surfactant content.

The oxidation speed of Ni is inherently low, therefore, insertion of even small quantities of Ni into Cu deposits effectively enhance its electrochemical properties. Moreover, a common application of 1,4-butynediol in industry is as an additive to increase the corrosion resistance of coatings [26]. Cu-Ni coatings were produced by conventional and sc-CO_2_ processes with various surfactant contents, and electrochemical properties were analyzed by PPS. From Section 3.2.1 we know that Cu content was the highest, therefore, pure Cu coatings were also analyzed for comparison. Representative polarization curves are shown in Figure 10 and Figure 11.

Figure 10 shows the polarization curve of a set of coatings produced with 2.5 g/L of surfactant content by the various processes. This specific surfactant content resulted in better mechanical properties on the previously discussed analyses, so enhanced electrochemical resistance was also expected. Pure Cu coatings produced by the conventional method displayed poor electrochemical resistance (−0.366 V, 2.50 µA/cm^2^), which promoted the motivation for this study. Cu-Ni coatings under the conventional process slightly improved the electrochemical resistance as hinted by the relatively higher E_corr_ and I_corr_ measured (−0.342 V, 6.89 µA/cm^2^); however, coatings produced by the sc-CO_2_ process evidently had even better electrochemical resistance (−0.198 V, 28.56 µA/cm^2^).

Figure 11 shows the polarization curves of a set of coatings produced with 3.0 g/L of surfactant content by the various processes. Similarly, pure Cu coatings by conventional process displayed the lowest electrochemical resistance (−0.368 V, 0.99 µA/cm^2^), Cu-Ni coatings by the conventional process displayed slightly better electrochemical resistance (−0.352 V, 5.42 µA/cm^2^) and Cu-Ni coatings by the sc-CO_2_ process displayed an even better electrochemical resistance (−0.257 V, 4.49 µA/cm^2^). Additionally, electrochemical resistance of coatings produced with 3.0 g/L surfactant content was inferior to those produced with 2.5 g/L surfactant content. Similar results were observed for analysis with lower or higher surfactant contents. This is attributed to the saturation condition discussed in the previous sections. For all cases, electrochemical resistance of Cu-Ni coatings exceeded that of pure Cu coatings regardless of the electroplating process used.

Moreover, a passivation region was observed for coatings produced by the conventional process but not for coatings produced by the sc-CO_2_ process. The passivation region is where I_corr_ kept relatively constant and low, which hints to the formation of barrier films causing drops in I_corr_. The breakdown of barrier films occurred when E_corr_ increased, but pitting corrosion becomes an issue from this point onward. Additionally, pinholes and defects significantly increase the possibility of localized corrosion, so the Cu-Ni coatings fabricated by the sc-CO_2_ process were expected to display higher electrochemical resistance compared to those produced by the conventional process. 

## 4. Conclusions

This work reports the fabrication of Cu-Ni alloy co-plating with 1,4-butynediol as a surfactant in sc-CO_2_ electroplating at low DC settings. Smaller grains resulted in brighter and smoother coatings produced by the sc-CO_2_ process. Saturation of the surfactant content was achieved at 2.5 g/L, a point where evident changes were observed for all of our experiments. 1,4-butynediol undergoes hydrogenation under the sc-CO_2_ environment and, thus, the surfactant could bind with free hydrogen ions in solution that normally reduced current efficiency. Lower or higher surfactant concentrations did not significantly affect Ni content on the coatings, nor the current efficiency of the process. The process described in this study effectively reduced pinholes normally caused by residual H^+^ on the coating’s surface through an effect similar to pulse plating and the hydrogen-binding capabilities of the surfactant. Moreover, application of the sc-CO_2_ process with added surfactant effectively reduced the coating roughness and the degree of internal stress when compared to the conventional process. A preferred crystal orientation of the Cu-Ni coatings was observed and remained in the (111) direction regardless of the process used, which verifies that surfactant content did not affect crystal orientation. Finally, coatings produced by the sc-CO_2_ process with surfactant displayed relatively less localized corrosion compared to the conventional process with surfactant due to a lower incidence of pinholes, as seen from the PPS experiments. 

This study has laid the groundwork for further research on alloy co-plating and future goals in our research group include more detailed descriptions on the deposition mechanisms due to the interactions of the added surfactant.

## Figures and Tables

**Table 1:** Electroplating parameters applied in this study.

Parameter	Conventional Electroplating	sc-CO_2_ Electroplating
Pressure (MPa)	0.1	15
Temperature (°C)	50	50
Agitation speed (rpm)	500	500
Current density (A/dm^2^)	2.5	2.5
Surfactant content (g/L)	2.0, 2.5, 3.0, 3.5, 4.0	2.0, 2.5, 3.0, 3.5, 4.0
Plating time (h)	1	1

**Table 2:** Parameters for the electrochemical experiment performed in this study.

Parameter	Setting
Exposed area of WE	1 cm^2^
Plated thickness	50 µm
OCP stabilization time	30 min
Scanning potential	Vs. reference SCE
PPS speed	1 mV/s
PPS range	Dependent on OCP: ±250 mV (Total scan range: 500 mV)
Corrosive solution	3.5% NaCl
Temperature	20 °C ± 2 °C
